# Molecular Research in Penile Cancer—Lessons Learned from the Past and Bright Horizons of the Future?

**DOI:** 10.3390/ijms141019494

**Published:** 2013-09-26

**Authors:** Chris Protzel, Philippe E. Spiess

**Affiliations:** 1 Department of Urology, University of Rostock, Rostock 18055, Germany; E-Mail: protzelc@med.uni-rostock.de; 2 Department of Genitourinary Oncology, Moffitt Cancer Center, Tampa, FL 33612, USA

**Keywords:** penile cancer, pathways, carcinogenesis, progression, metastasis

## Abstract

Penile cancer is a rare tumor. There is a limited understanding of the biological mediators of prognostic and therapeutic importance in penile cancer. However, there exists some fundamental understanding of the major pathways involved in the development of penile pre-neoplastic lesions and neoplasms. The aim of the present review is to highlight our current state of molecular knowledge in penile cancer to foster the necessary tools for researchers to pave major advancements in our current treatment paradigms and cancer specific outcomes.

## 1. Introduction

Molecular research has become a fundamental tool for our in-depth understanding into carcinogenesis, with its downstream implications in cancer therapeutics. By targeting important cancer molecular pathways, we have now embarked into the era of targeted drug therapy, which is critical in our armamentarium against often lethal medical conditions. Unfortunately, this remarkable progress in the drug design and discovery has not encompassed all tumor types and/or underlying histologies.

Penile cancer is a rare tumor entity within the developed western world [1]. This bears two major problems for the patients and urologists alike. First, there is only limited fundamental biological and clinical knowledge on this tumor entity since most centers of excellence treat only a limited number of patients annually. In consequence, our abilities to extensively study this malignancy remains somewhat hindered. Second, due to the limited number of patients and tumor samples at single centers, there is only limited tissue material accessible for molecular and translational studies at most individual treatment facilities. This second point however has led to efforts in organizing international research collaborations, which fosters great promise for future progress.

The first step to advancing our scientific understanding of this tumor phenotype is the identification of the major pathways and mediators involved in the development of penile pre-neoplastic lesions and neoplasms (see Table 1). Cancer cells bear remarkable individual abilities which enable them to survive and proliferate within a milieu of a perfect human immune defense system. However, these special capabilities of cancer cells are also potential targets for further therapeutic approaches. Therefore, an understanding of these mechanisms is the first step to the generation of successful targeted therapy in penile cancer.

## 2. What Do We Focus on?

The three key mechanisms of cancer progression are:

1. Carcinogenesis (escape from apoptosis, tumor suppressor genes, and immune defense mechanisms),
2. Tumor progression (invasion, transformation),
3. Metastatic spread and metastatic seeding (resistance to environmental influences *i.e*., chemoresistance).

**Figure 1:**
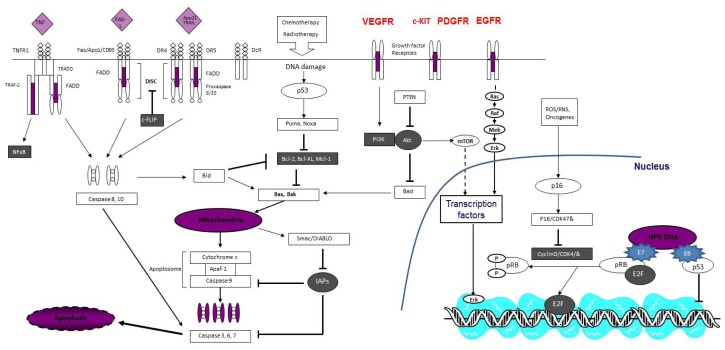
Schematic diagram illustrating molecular pathways implicated in penile cancer (diagram has been modified and replicated with the explicit permission of *Expert Opin. Emerg. Drugs*; “Emerging apoptosis agonists for bladder cancer”; Protzel, C.; Hakenberg, O.W. **2009**, *14*, 607–618).

Our review article on molecular research in penile cancer will highlight our current state of knowledge into the molecular mechanisms believed to be fundamental in penile cancer providing a framework of where future research endeavors will likely be fruitful in impacting the natural history and treatment approaches for this poorly understood tumor phenotype (see Figure 1).

## 3. Molecular Mechanisms of Penile Cancer Progression

### 3.1. Carcinogenesis

Carcinogenesis is a multifactorial process of transformation of normal tissue into abnormal continuously proliferating tumor cells. The detailed mechanistic steps of this process are still unknown, but however they are known to include: genomic instability, DNA damage, resisting cell death, immortalization and immune-escape which are now considered “hallmarks” of carcinogenesis [2]. There is reasonable data to support the role of these biological processes specifically in penile carcinoma.

Since chronic inflammation is believed to be one of the most important risk factors for penile cancer, mediators of inflammation may play a special role in penile carcinogenesis [3]. Reactive oxygen/nitrogen species (ROS/RNS) are produced by inflammatory cells to fight infectious agents, but they can also damage the DNA of cells within the surrounding tissue. There are special cell control mechanisms termed tumor suppressor genes which react to DNA damage inducing cell arrest and/or apoptosis [4,5]. A key tumor suppressor gene for ROS/RNS damage is *p16*. Activation of p16 causes cell arrest by the inhibition of cyclin D—a cyclin dependent kinase mediated release of E2F. Since loss of heterozygosity has been shown to be frequently found in the *p16* gene, it is postulated that this pathway may play a critical role in penile carcinogenesis particularly in the clinical context of chronic inflammation [6]. Other key mediators in inflammation induced carcinogenesis are cyclooxygenase-2 (COX-2) and prostaglandin E2 (PGE2). Overexpression of COX-2 causes an overproduction of prostaglandins and thromboxans, with PGE2 playing a pivotal role in proliferation, angiogenesis, and activation of epidermal growth factor receptor [5]. PGE2 also activates β-catenin-T-cell factor (co-function for replicative potential and immortalization) and PI3K (responsible for cell migration and invasion) [5,7]. COX-2 has also been shown to be strongly expressed in penile carcinoma [8]. In addition, anti-inflammatory/pro-resolving mediators have become a notable target of tumor therapeutic approaches being developed [5]. Specific risk factors for penile cancer seem to be obviously associated with these pathways. Chronic inflammation like balanoposthitis and lichen sclerosus have been shown as the most important risk factors in several studies [9]. Phimosis is mostly associated with chronic inflammation and therefore another risk factor. The role of tobacco smoke as a risk factor remains highly debatable, since a number of studies did not show a strong association with this endpoint [3,9]. Nevertheless a potential role in penile carcinogenesis is quite feasible as the metabolism of *N*-Nitrosodiethylanine has been shown in sebaceous glands and within urine contaminants among cigarette smokers [10]. Prolonged retention of these potential carcinogens under the foreskin may explain this cancer de-differentiation. Circumcision in childhood has clearly been shown as a protective factor to minimize this risk of penile cancer. Furthermore, the higher viral infection rate within uncircumcised males could explain the cancer preventative impact of circumcision independent from its underlying mechanism of minimizing chronic inflammation [11]. Since early sexual contacts, promiscuity, and oral sexual practices are also known risk factors for penile cancer, the potential role of viral infection in inducing carcinogenesis seems quite biologically plausible [9].

Oncogenic viruses are believed to play an important role in several tumor types. Cervical cancer is known to be associated with high risk papilloma virus (HPV) serotypes (*i.e*., serotype 16, 18, 31, 33) which have similarly been strongly associated with penile cancer [12,13]. HPV DNA was found in an estimated 30% to 50% of conventional squamous cell carcinomas of the penis [6,13,14]. Therefore, the role of HPV in penile carcinogenesis is of significant interest although poorly studied up until this point. Inactivation of p53 by HPV-E6 plays a key role in HPV oncogene associated carcinogenesis. Strong expression of p53 leads to an inhibition of the cell cycle by the p21/Retinoblastoma (Rb) cascade [2]. This negative cell cycle regulation is also disturbed by HPV oncogenes, namely E7, which binds and inactivates Rb [2]. Recent studies have shown that HPV-associated alterations of tumor suppressor genes *p53/pRb/p16* are only found in a limited number of penile cancer cases particularly basaloid and warty subtypes of penile cancer [6].

Nevertheless, tumor suppressor gene alterations are a major area of focus in penile cancer translational research. A relevant number of genetic alterations in tumor suppressor genes were shown to be fundamentally pertinent in penile cancer. Loss of heterozygosity (LOH) was frequently found on chromosomes 2q, 6p, 8q, 9p, 12q, and 17p13 suggesting the presence of important tumor suppressor genes in these respective regions [15]. LOH in the chromosomal loci 6p22–23 was significantly associated with a poor prognosis among penile cancer patients [15]. Therefore, an important tumor suppressor gene is believed to be present within this region. Genetic imbalances have been shown in several other chromosomal regions within penile carcinoma. Gains in copy number are frequently reported within the 8q24 chromosomal region [16]. The proto-oncogene *MYC* is located within this region and several studies among a number of tumor types have demonstrated the insertion of HPV16 DNA within this region. The inserted HPV DNA led to an overamplification of the *MYC* proto-oncogene in several tumor cell lines including a penile cancer specific cell line [17]. Masferrer *et al*. have furthermore reported MYC gains and MYC overexpression in human penile carcinomas [18]. In accordance to previous reports about a HPV triggered MYC activation, these authors were able to show an association between HPV detection and strong MYC expression. Additionally, increases in MYC expression was associated with tumor progression and poor cancer-specific outcome within this patient population.

Resisting cell death by evading the apoptotic machinery is another important hallmark of cancer [2]. Unfortunately, there is only very limited data about the key regulators of the intrinsic and extrinsic pathway of apoptosis in penile cancer. This is unfortunate as the pharmacological interaction between apoptotic pathways are increasing being recognized in cancer therapeutics [19]. Pro-apoptotic receptors, activated by members of the greater cytokine TNF superfamily (e.g., Apo2L/TRAIL), play a key role for the extrinsic pathway [20]. Genetic instability was shown for the 8p21–21 loci in penile cancer, where the pro-apoptotic TNF receptors *DR4* and *DR5* are located [21]. Data concerning expression of caspases are similarly limited. The tumor suppressor p53 plays a central role in the activation of the intrinsic pathway by activation of Noxa and Puma in response to DNA and cell damage. Both inhibit the anti-apoptotic key player bcl-2 [20]. Therefore, alteration of p53 frequently found in penile cancer decreases the apoptotic potential of tumor cells [22,23]. In line with the alteration of p53, an activation of bcl-2 and weak expression of bax which is negatively regulated by bcl-2 was found in a small pilot study of penile cancer patients [24].

The immortalization and unlimited replicative potential of cancer cells are also important factors for the limitless progression of malignant tumors. Tumor cells have to overcome senescence which is usually initiated in cells after a given number of cell cycle divisions. Telomerases are very important for cells to overcome the regulated process of senescense [2]. Telomerases are DNA polymerases which add telomere repeats to the DNA as part of senescence. Telomeres are multiple tandem hexanucleotide repeats, which shorten progressively with each cell division in non-immortalized cells [25]. Telomerase activity is significantly increased in many tumor entities while it is nearly completely missing in normal and premalignant cells [25]. Alves *et al*. showed telomerase activity in up to 85% of invasive penile carcinomas [26].

In conclusion penile cancer shares many similarities with other squamous cell carcinomas (SCC) e.g., head and neck, esophageal, and cervix SCC. The role of HPV DNA seems not as important in penile cancer as it is in cervical cancer, since only 30%–50% of the penile SCC show HPV DNA (although different in warty and basaloid subtypes). While chronic inflammation seems to be the most important pathway of carcinogenesis in penile cancer, penile cancer shares the activation of COX-2, PGE2 as well as EGFR with most of the other SCC tumor phenotypes. This fact creates unique opportunities for a better understanding and treatment of penile cancer. Targeted drugs successfully used in other SCC organ sites may serve a potential role in penile cancer as shown in the preliminary reports employing EGFR antibodies (discussed further in the section below). Similarly, it is believed that advances in our understanding of the biological pathways of carcinogenesis in SCC (e.g., miRNAs) in other organ sites may ultimately unravel our better appreciation of the fundamental molecular basis of penile cancer in the years to come.

### 3.2. Progression and Invasion

Proliferation is an important characteristic of tumors; it causes local tumor growth and dissemination of cancer cells [2]. Therefore, proliferation markers have been used to predict the prognosis and metastatic capability of malignant tumors. The proliferation marker Ki67 has been shown to be highly expressed in more aggressive penile carcinomas [27]. The strong expression of Ki67 was associated with a higher tendency of metastasis and poor survival within clinical studies [27]. The proliferation marker PCNA was also examined in penile cancer. Expression of PCNA was significantly associated with lymph node metastases but failed to show prognostic significance with regards to cancer-specific survival [22].

Progression caused by constant proliferative signaling is another important characteristic of malignant tumors. Growth factors play a major role in this biological process. Tumor cells produce growth factors and express corresponding receptors for them (autocrine stimulation), tumor cells stimulate surrounding stroma cells to produce growth factors (paracrine stimulation), and they are able to sustain growth stimulation by the continuous activation of downstream pathways of growth factor receptors (*i.e*., activating a mutation of the B-Raf protein) [2].

Overexpression of EGFR is frequently found in various tumor entities. EGFR is an ERBB receptor tyrosine kinase. Epidermal growth factor receptor (EGFR) is strongly expressed in penile carcinoma tissue whereby suggesting it may play an important role in penile carcinogenesis [28]. The EGFR pathway is likely to serve as an important therapeutic target in coming years, with panitumumab (an EGFR antibody) shown to exhibit some biological activity in the few cases of advanced penile cancer patients it was employed [29,30].

Activation of EGFR by epidermal growth factor (EGF) or transforming growth factor-α (TGFα) leads to an activation of an intracellular tyrosine kinase unit which induces several proliferative pathways. In a recent study, the *KRAS-BRAF* pathway, which is a major EGFR dependent pathway, was examined in penile carcinomas [31]. In 150 cases, only one *KRAS* and no *BRAF* mutations were found. Since the effect of anti-EGFR treatment highly depends on intact *KRAS*, these findings are very meaningful for penile cancer treatment. They also found the potential tumor suppressor RASSF1A (RAS-association domain family 1A) downregulated in penile carcinomas, showing the important role of the EGFR-RAS signaling pathway in the pathogenesis of penile cancer [31]. Other members of the ERBB family include ERBB2-HER-2, ERBB3, and ERBB4. While HER-2 was not detected in penile carcinomas, HER-3 and HER-4 have been shown to be associated with penile carcinogenesis. A second major growth receptor/tyrosine kinase signaling pathway is the PI3K/PTEN/AKT pathway, which is frequently altered in malignant tumors [32]. Andersson *et al*. showed mutations of *PIK3C*A (catalytic subunit of PI3K) in 29% of the examined tumors [33]. Allelic losses in *PTEN* have been also reported.

Other important biological processes of tumor progression include invasion and metastatic spread which are believed to be highly correlated to changes within the microenvironment of the tumor and the epithelial-mesenchymal transition (EMT). The essential part of the invasion is the breakdown of the cell-to-cell adhesion in the tumor invasion front [2,34]. This enables tumor cells to invade through the basement membrane. Since E-cadherin is a key player in the mediation of intercellular junctions, E-cadherin has to be downregulated in the process of EMT and invasion [34]. EMT inducing factors like Snail, Slug, Twist, ZEB1 and ZEB2 decrease the expression of E-cadherin [34]. There is corroborative data that miRNAs play an important role in the regulation of EMT. Members of the miR-200 family suppress EMT [35]. They are often downregulated in invasive tumors for instance in head and neck squamous cell carcinomas (HNSCC) [36]. In contrast, increased expression of miR-21 is associated with EMT and tumor progression [35]. The tumor cells in the invasion front reacquire several properties evident during embryonic morphogenesis and wound healing corroborated with the increased levels of mesenchymal markers like vimentin, *N*-cadherin, fibronectin, and Tenascin C which are seen [34]. Activation of matrix metalloproteinases (MMPs) by ZEB or Tenascin is another characteristic in the invasion front of tumors [34]. Until now, there is only limited data about the role of EMT in penile carcinoma. Data pertaining to the expression of EMT regulators and miRNAs as well as their role in tumor progression is greatly needed. Nevertheless, there is some preliminary data supporting the essential role of EMT in penile cancer, with the work of Campos *et al*. reporting that the decreased expression of E-cadherin in penile cancer was associated with tumor progression [37]. The strong expression of Tenascin in the invasion front was similarly shown in penile carcinoma. Campos *et al*. and Zhu *et al*. further reported an increased expression of MMPs within such tumors [37,38].

The process of invasion and EMT is also characterized by several typical changes in protein expression within the penile cancer tumor cells at the invasion front. Cell surface proteins like Annexin (ANX I closely related to EGFR), Fibronectin are highly expressed in this cellular process [39]. The strong expression of glucose transporter 1 illustrates the high metabolic needs of such invading tumor cells [40].

### 3.3. Metastasis and Resistance to Environmental Influences

Penetration of the basement membrane is the key step in metastatic progression. Tumor cells initiate a specific interaction with the surrounding tissue, the microenvironment, as stromal-epithelial interactions [2]. The tumor microenvironment plays a major role in enhancing tumor cell infiltration by recruited macrophages (tumor associated macrophages abbreviated as TAM). They increase tumor cell mobility and angiogenesis. Neoangiogenesis is very important for the intravasation of tumor cells [41,42]. Tumor cells have to cross the pericyte-endothelial cell barrier of the microvessels. TGFβ and TAM play an important role in this cellular process. Specific tumor vessels stimulated by VEGF are easier to invade, since they are characterized by the loss of pericyte coverage and larger leaks compared to normal vessels [42]. Expression of VEGF was shown in penile carcinomas by de Paula *et al*. who demonstrated that VEGF C is an independent prognostic factor for metastatic progression [43].

The intravasation of tumor cells is followed by their ability to survive within the circulation. Unique phenotypic characteristics are once again needed for this metastatic propensity to be successful. At present, there is no data about the role of circulating tumor cells (CTC) for penile cancer. Since tumor cells have to survive without ECM components, they have to activate special metabolic pathways [42]. The re-expression of embryonal glucose transporters in penile carcinoma cells is believed to be important in this regard [40]. The metastasis suppressor gene *KAI1* seems to play an important role in the prevention of tumor cell circulation. KAI1 leads to a Duffy anti-gene receptor for chemokine (DARC) associated binding and destruction of tumor cells [44]. Loss of KAI1 expression leads to free circulation and metastatic seeding. Loss of KAI1 expression was significantly associated with the occurrence of lymph node metastasis and an overall poor prognosis among patients with penile cancer [45]. Once entry in the circulatory system is successful, metastatic tumor cells are able to reach special organ sites (*i.e*., preferred sites for metastatic cancer dissemination). Carcinomas of different origins show a tissue tropism, with unique cellular mutations and genetic expression profiles characterizing metastatic cells disseminating to bone or lung sites [42]. Although several specific chromosomal aberrations have been shown in penile cancer metastasis, there is a great necessity to better characterize these unique features of penile cancer cells at distant metastatic sites [15]. The process of extravasation is again characterized by the activation of several enzymes such as MMPs, COX, and angiopoetin like-4 [2,42].

There is recent data suggesting the establishment of a “pre-metastatic niche”. In these niches, micrometastasis may acquire further capabilities and specific mutations which enable them to start the growth of macrometastases. TAM plays again an important role in the modification of this special microenvironment [42]. In this process, the tumor cells undergo MET (mesenchymal-epithelial transformation). This process is again controlled by miRNAs, e.g., re-expression of miR 200 family [35]. Strong expression of nm23 seems to suppress these cellular processes [42]. Since loss of nm23H1 expression has been shown to be associated with metastasis in penile cancer and re-expression of nm23 showed to exhibit anti-metastatic effects *in vitro*, there may be important therapeutic implications of nm23 in future drug discovery efforts [46,47].

The micrometastastatic niches are also proposed as a mechanism of survival against systemic chemotherapy [48]. There are several proposed mechanisms of resistance against antineoplastic agents. These can either be primary or acquired resistance. Frequent mechanisms are specific to the sequential steps of drug metabolism including uptake, efflux and detoxification, enhanced DNA repair, dysregulation or absence of apoptotic proteins, and modification or mutations of drug targets [48]. Excision repair cross-complementary group 1 (ERCC1) is a key molecule in nucleotide excision repair [49]. It has been examined in several tumor entities and seems to be useful as potential predictor for cisplatin resistance. Negative or weak expression of ERCC1 was associated with better systemic chemotherapeutic response in HNSCC, bladder cancer and non small cell lung cancer (NSCLC) [49–51]. Polymorphisms of repair genes *ERCC1* and *XRCC1* were also associated with better response [51]. Strong expression of RASSF1A and weak expression of ERCC1 were predictors for response to docetaxel and cisplatin systemic chemotherapy [49]. Micro RNAs seem to also play an important role in chemoresistance. High expression of miR-200c in combination with cytoplasmic expression of HuR (RNA-binding protein) was found to be associated with resistance to paclitaxel in ovarian cancer [52]. Until now, there is no data about predictors of treatment response in penile cancer, but further explorative and translational studies may pave the way to individualized treatment approaches as well as novel multifactorial approaches employing specific genetic knockout strategies to ERCC1 or miRNAs.

The role of primary chemo-and radiation resistant CTC has recently been demonstrated. There is a special subclass within the heterogeneous class of intratumoral cancer cells, which are characterized by their ability to seed new tumors upon inoculation within recipient host tissue as was shown in a murine model [53]. There is data to support the creation of new tumors or metastatic clones with up to 107 tumor cells by a single CSC [54]. CSC show differing biomarker profiles and have an unlimited potential in terms of their number of cellular divisions. A targeted treatment of CSC seems to be a mechanism by which refractory tumors can overcome systemic therapies [55]. CSC have not been described in penile cancer as of yet but however are described for the more thoroughly investigated HNSCC [56]. Therefore, the search for CSC in penile carcinoma offers great promise as a novel and highly anticipated therapeutic approach.

## 4. Conclusions

The molecular mechanisms of carcinogenesis and tumor progression are important therapeutic targets of present and future studies in penile cancer. One of the most important components of successful research efforts and improved understanding in penile cancer are the establishment of international and multi-institutional penile cancer study collaborations. The identification of mechanisms of early invasion and metastatic spread offers the possibility of detection of penile neoplasms which are best suited with early aggressive therapy as well as the design and discovery of new small molecule target agents in the not too distant future. Since there is mounting evidence as to the importance of miRNAs and CSCs in tumor progression and chemoresistance, the role of these potential therapeutic targets should be actively pursued in current penile cancer research efforts. Failure to do so may in fact result in a missed opportunity to optimize patient care within this patient cohort.

## Figures and Tables

**Table 1:** Molecular changes reported for penile carcinomas.

Carcinogenesis	Proliferation/Invasion	Metastases
**Inflammation**	**Growth factors/receptors**	**Metastases suppressor genes**
COX-2	EGFR	*KAI1*
PGE-2	HER-3/HER-4	*Nm23H1*
**Tumor suppressor genes**	VEGF	
*p53*	PI3K/PTEN/AKT	
*p16*	**EMT**	
*PTEN*	MMP2/MMP9	
**Oncogenes**	E-cadherin	
*HPV E6/E7*	TenascinC	
*MYC*	Annexins	
**Apoptosis/cell death**	Glut1	
DR4/DR5		
Bcl-2/BAX		
*p53*		
Telomerases		
